# Consensus Conference Series on Dysglycemia-Based Chronic Disease (DBCD) in Latin America: The Chilean Transculturalization

**DOI:** 10.5334/aogh.5370

**Published:** 2026-07-13

**Authors:** Ramfis Nieto-Martinez, Carlos Grekin, Diana De Oliveira-Gomes, Juan P. Gonzalez-Rivas, Sandra Lopez-Arana, Jeffrey I. Mechanick

**Affiliations:** 1 Precision Care Clinic Corp, Saint Cloud, Florida, USA; 2 Departments of Global Health and Population, Harvard TH Chan School of Public Health, Boston, MA, USA; 3 Foundation for Clinic, Public Health, and Epidemiology Research of Venezuela (FISPEVEN INC), Caracas, Venezuela; 4 Nutrition and Diabetes Unit, Clínica Red Salud Vitacura, Santiago, Chile; 5 Nutrition and Diabetes Service, Santiago Military Hospital, Santiago, Chile; 6 University of the Andes, Santiago, Chile; 7 Division of Cardiovascular Medicine, Brigham and Women’s Hospital, Harvard Medical School, Boston, MA, USA; 8 University Center for Health Sciences – HM Hospitals (CUHMED), Camilo Jose Cela University, Madrid, Spain; 9 HM Hospitals Health Research Institute, Madrid, Spain; 10 School of Nutrition and Dietetics, Faculty of Medicine & Health, Finis Terrae University, Santiago, Chile; 11 The Marie-Josee and Henry R. Kravis Center for Cardiovascular Health at Mount Sinai Fuster Heart Hospital, Icahn School of Medicine at Mount Sinai, New York, New York, USA

**Keywords:** Chile, diabetes, dysglycemia-based chronic disease, transculturalization

## Abstract

*Objective:* The MEchanick Transculturalization Research and Innovation ConSortium/Bernard Lown Scholars in Cardiovascular Health Program Consensus Conference on Dysglycemia-Based Chronic Disease (DBCD) Transculturalization in Chile convened on November 20, 2023, in Santiago, Chile. The conference generated affirmed and emergent concepts, key strategies, and specific implementation tactics to improve type 2 diabetes (T2D) care in Chile.

*Findings:* Important affirmed concepts included: (1) implementing a comprehensive approach to T2D management beyond glycemic control; (2) addressing unique challenges for early detection and treatment of T2D; and (3) applying expanded roles of telemedicine. Important emergent concepts included: (1) adopting transculturalized chronic care models such as DBCD; (2) recognizing prediabetes as a critical DBCD target to prevent T2D and T2D complications, especially cardiovascular disease; and (3) implementation of the DBCD model for individual and population health. Key strategies included: (1) validation of culturally adapted T2D risk assessment tools; (2) integration of social determinants of health (SDOH) and ethnocultural factors into DBCD care strategies/tactics; and (3) promotion of equity in healthcare access for all people comprising diverse populations. Finally, specific implementation tactics included: (1) focusing on patient-centered public policies; (2) ensuring access to effective treatments; and (3) using culturally relevant resources for education and prevention. When coordinated, these strategies and tactics mitigate DBCD progression, thereby enhancing healthcare outcomes.

*Conclusions and recommendations:* Expert consensus emphasizes the need for a comprehensive approach to T2D management in Chile, leveraging transculturalized lifestyle medicine, validated risk assessment tools, SDOH, and patient-centered public policies. This process should begin with incorporating eHealth technologies, validation studies, and then translation into clinical practice guidelines. As this templated methodology is applied to other regions of the world, the resulting compendium of concepts, strategies, and tactics can foment a more effective preventive health culture and optimize DBCD care across the ethnocultural spectrum.

## Executive Summary

Affirmed and emergent concepts from five critical questions are summarized in Table 1.

**Table 1:** Summary of Affirmed and Emergent Concepts Based on Five Critical Questions.

QUESTION	AFFIRMED CONCEPTS	EMERGENT CONCEPTS
*Question 1* What is a comprehensive approach to T2D care?	The T2D approach must be comprehensive (addressing modifiable risk factors that impel DBCD and lead to complications) and provided through the DBCD chronic care model.	DBCD broadens traditional T2D chronic care models to include relevant social, environmental, and cultural factors and therefore should be used in Chile.
*Question 2*. What is prediabetes and is it important to diagnose?	Although it is associated with the presence of complications, prediabetes is considered a predisease and not a disease.	Prediabetes, as stage 2 of the DBCD model, is an actionable condition. Prediabetes should be prevented, and if identified, treated.
	The Prediabetes definition is based on glycemia cutoffs associated with a stipulated statistical risk for T2D.	Lower cutoffs for prediabetes should be used for Latin American populations. Treatment goals for prediabetes are based on cardiorenal, neuropathic, and cognitive impairment outcomes.
*Question 3* Is the diagnosis of DBCD useful in Chile and should screening and case finding be recommended?	Detection and treatment of DBCD is typically late (when T2D is diagnosed) and needs to occur earlier.	Leveraging the DBCD model will expose opportunities for earlier detection (i.e., before actual T2D with/without complications) and improve outcomes.
	FINDRISC is a useful T2D risk screening tool globally that is validated for the Latino population and may be useful for Chile.	FINDRISC should be adopted in Chile as a T2D screening tool and implemented via eHealth technology over social networks.
	Dysglycemia is not associated with increased adiposity in certain Latino populations.	OGTT should be performed in any Chilean patient with a cardiometabolic risk factor regardless of age or BMI.
*Question 4* What are the key features of a Chilean transcultural approach to DBCD?	Current chronic care models for T2D do not specifically consider transcultural factors though they should be considered for more precise clinical management.	The transculturalized DBCD model, incorporating concepts from the validated tDNA in the region should be implemented as part of T2D care in Chile.
	Migrant population health imposes a challenge for Chilean health systems and urgently needs to be addressed.	Vulnerable migrant populations should be included in transculturalized DBCD recommendations in Chile.
*Question 5* What are the core Chilean lifestyle interventions for each DBCD stage?	Prioritizing lifestyle medicine in T2D care is a significant practice gap that can be addressed by implementing the DBCD model.	Specific Chilean transcultural adaptations of lifestyle interventions pertaining to each DBCD stage should be investigated, taught, and implemented.
	The Coronavirus disease 2019 pandemic increased awareness of this practice gap and accelerated the use of telemedicine modalities.	eHealth technologies can facilitate this process of transcultural lifestyle medicine in T2D care in Chile.

*Abbreviations:* A1C—glycated hemoglobin A1c; DBCD—dysglycemia-based chronic disease; eHealth—electronic health; FBG—fasting blood glucose; FINDRISC—Finnish Diabetes Risk Score; OGTT—oral glucose tolerance test; T2D—type 2 diabetes; tDNA—transcultural diabetes nutrition algorithm; WC—waist circumference.

### Affirmed concepts

- A comprehensive approach to type 2 diabetes (T2D) management extends beyond glycemic control, addressing multifaceted pathophysiology (e.g., abnormal adiposity, insulin resistance, and inflammation), as well as downstream complications and long-term health impacts [1]. Thus, T2D care should be provided through a chronic care model [2].
- Prediabetes represents a critical stage for early intervention to prevent T2D and T2D complications, especially cardiovascular disease (CVD).
- Delayed T2D detection and treatment results from significant knowledge/practice gaps, as diagnosis often occurs at advanced stages when complications and increased clinical/economic burdens have already developed.
- The Finnish Diabetes Risk Score (FINDRISC) tool has gained global adoption for T2D risk screening [3], including a version validated for the Latino population with waist circumference (WC) adaptations [4], though this has yet to be incorporated into dedicated Chilean clinical practice guidelines.
- Most available CVD risk formulas were designed for, and validated in, populations from high-income countries, with limited representation of diverse ethnocultural groups.
- Clinical practice guidelines recommend dysglycemia case-finding using fasting glucose, hemoglobin A1c (A1C), and an oral glucose tolerance test (OGTT) in patients over 10 years old who are overweight [5], yet dysglycemia is not consistently linked to excess adiposity in some Latino populations [6].
- Current diagnostic and treatment models in Chile do not account for ethnocultural diversity, especially in lifestyle, leading to significant practice gaps in dysglycemia care.
- The health of migrant populations presents an additional challenge for the Chilean healthcare system, requiring adaptable and inclusive approaches.
- Although lifestyle interventions are recognized as essential to management and remission of T2D, their integration into healthcare infrastructures and economic models and the creation of successful lifestyle medicine centers has been limited.
- The expansion of telemedicine, accelerated by the Coronavirus disease 2019 pandemic, has reshaped healthcare delivery, offering new opportunities for T2D prevention and management.

### Emergent concepts

- The Chilean healthcare systems should adopt chronic care models, such as dysglycemia-based chronic disease (DBCD), tailoring to local needs and ensuring that they address the complexities of dysglycemia and related conditions.
- The DBCD model broadens the scope of T2D care in Chile by incorporating social, environmental, behavioral, and ethnocultural factors into a comprehensive approach. Addressing all four stages of the dysglycemia spectrum (DBCD stage 1 [DBCD-1] risk; DBCD-2 predisease/prediabetes; DBCD-3 disease/T2D; and DBCD-4 T2D complications) can lead to earlier detection of pathophysiology, lower risk for complications, and improved outcomes [7].
- Prediabetes is an actionable state that should be detected and treated early to prevent progression to later DBCD stages, which can reduce clinical/economic burdens in the Chilean population.
- Lower glycemic cutoffs have been proposed for Latino populations to better account for region-specific risks of T2D, and this is highly relevant to chronic disease care models in Chile [8].
- Treatment goals should prioritize cardio-renal outcomes and improve detection of neuropathy and cognitive impairment in T2D management in Chile.
- FINDRISC should be used as a T2D screening tool in Chile [9], leveraging eHealth technologies to identify high-risk populations [10].
- GLOBORISK is a cardiovascular disease risk equation that has been recalibrated with routinely available data to generate country-specific risk chart, including a Chilean version [11].
- Performing an OGTT in patients in Chile with one cardiometabolic risk factor may enhance early detection of prediabetes and T2D, particularly in those with a normal and low body mass index [12].
- The transcultural adaptation and implementation of the DBCD model in Chile can be guided by the transcultural diabetes nutrition algorithm (tDNA) [13], which has been validated in Mexico [14], Brazil [15], and Venezuela [16], and can be expanded to other countries in the region.
- Special attention should be given to incorporating vulnerable migrant populations in Chile into DBCD recommendations and care strategies.
- Culturally adapted lifestyle interventions are essential to mitigate DBCD progression in Chile.
- New eHealth technologies offer opportunities to expand care provision, enabling broader access to medical and lifestyle interventions for managing DBCD in Chile.

## Introduction

### Diabetes care in chile

Data from the 2016–2017 Chilean National Survey reported a diabetes prevalence of 12.3% [17]. As of 2024, Chile has a population of approximately 19 million, with 87% residing in urban areas. The Chilean population is aging, with a median age of 35 years, driven by rising life expectancy and declining birth rates. Most Chileans are of Mestizo descent, a mixture of indigenous (43.2%) and European ancestry (54.4%) [18], with significant European communities of Spanish, Italian, German, and British origin. Indigenous peoples, mainly the Mapuche, comprise about 10% of the population, primarily in the south-central region. By 2022, Chile had 1,625,074 immigrants, accounting for 8.8% of the country’s total population [19].

The Chilean healthcare hybrid system comprises: (1) public healthcare by the National Health Fund (Fondo Nacional de Salud; FONASA), which is funded through taxation, covering around 80% of the population, and offering services at public facilities; (2) private insurance and healthcare professionals (HCPs) through Institutions of Social Security of Health (Instituciones de Salud Previsional; ISAPRES), offering higher-quality services for those who can afford them [20]; (3) Universal Access Plan to Explicit Guarantees (Plan de Acceso Universal a Garantías Explícitas; AUGE), which ensures timely care of high-priority conditions for both public and private beneficiaries; and (4) healthcare regulation overseen by the Ministry of Health, which is responsible for policies and standards [21]. Agreements within this healthcare system reduce the waiting list of patients in the public domain. As with healthcare systems in other countries, challenges include disparities, long waiting times, unequal coverage, and most relevant here, limited inclusion of lifestyle medicine. Besides, Chile has become a key destination for migrants from South America and the Caribbean, particularly Venezuela and Haiti. Regardless of their migratory status, they are entitled to healthcare. Navigating the Chilean healthcare system involves enrolling in FONASA and establishing a connection with a Family Health Center (Centro de Salud Familiar, CESFAM). CESFAM is a network of establishments providing comprehensive care to individuals and families, prioritizing disease prevention and the promotion of well-being.

Although screening tools and glycemic thresholds exist, they have not been systematically recalibrated for Chile’s heterogeneous population. Migrant health and social determinants of health (SDOH) also remain under-integrated into traditional care models. Beyond Chile’s genetic, ethnic, cultural, religious, and biological particularities, environmental exposures should be incorporated into a structured, stage-based framework integrating prevention, early detection, and complication management to mitigate the growing type 2 diabetes (T2D) burden.

### The dysglycemia-based chronic disease model

The dysglycemia-based chronic disease (DBCD) model conceptualizes dysglycemia as a progressive continuum encompassing risk, predisease, overt disease, and complications, providing a preventive template for cardiometabolic care (i.e., mitigation of chronic disease progression to subsequent stages or even reversion to antecedent stages). Specifically, the four distinct stages of DBCD are: stage 1 (risk) characterized by primary drivers (genetic, exposome/environmental, and resulting lifestyle behaviors) leading to secondary pathophysiological drivers such as insulin resistance; stage 2 (predisease) characterized by early/subtle beta-cell defects leading to mild hyperglycemia and representing prediabetes; stage 3 (disease) characterized by more significant β-cell defects leading to moderate–severe hyperglycemia and meeting diagnostic criteria for T2D; and stage 4 (complications) characterized by microvascular and macrovascular disease [7]. The way in which primary and secondary drivers interact to impel DBCD through the four stages is further modulated by prevailing SDOH and transcultural factors [22]. Thus, embracing the DBCD model offers an opportunity to implement culturally tailored and structured preventive care strategies within the Chilean population. Such an approach would allow for effective intervention at early chronic disease stages, reducing both clinical and economic burdens of dysglycemia.

### The transcultural aspect

**Figure 1:**
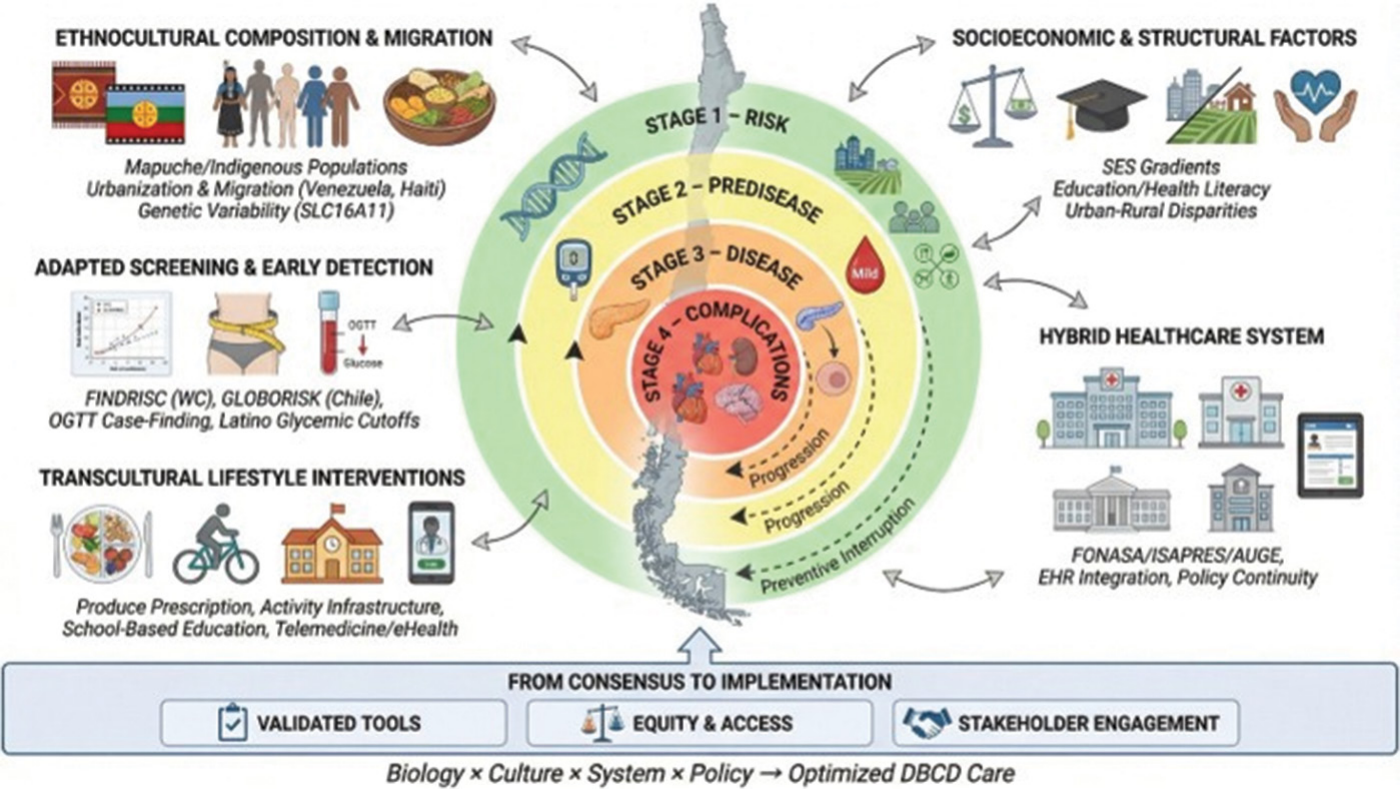
A multilevel framework for the prevention and management of the dysglycemia-based chronic disease (DBCD) in Chile, depicting how biological, cultural, healthcare system, and policy factors interact across four stages of disease progression with the goal of preventive interruption at each stage. *Abbreviations:* DBCD, Diabetes and Cardiometabolic Disease; FINDRISC, Finnish Diabetes Risk Score; WC, Waist Circumference; OGTT, Oral Glucose Tolerance Test; SLC16A11, Solute Carrier Family 16 Member 11; SES, Socioeconomic Status; FONASA, Fondo Nacional de Salud; ISAPRES, Instituciones de Salud Previsional; AUGE, Acceso Universal con Garantías Explícitas; EHR, Electronic Health Record; eHealth, Electronic Health.

A key step in the adoption of the DBCD model in Chile is its transcultural adaptation to ensure that it aligns with the biological, SDOH, and cultural aspects of the Chilean population. Transculturalization refers to the adaptation of evidence-based clinical recommendations from a source ethnocultural population to a target ethnocultural population, while preserving the integrity of each culture. Transculturalization requires the involvement of experts from both the source and target populations with structured, interactive, and iterative exchanges [23]. The transcultural Diabetes Nutrition Algorithm (tDNA) is a validated methodology for transculturally adapting evidence-based recommendations in the diabetes nutrition space to different ethnocultural groups and populations and was used in this consensus conference. According to the tDNA protocol, thought leaders would compile scientific evidence from multiple source populations (e.g., U.S., Canada, Europe, Latin American, and Asia), synthesize a DBCD model, select a target population with a distinct culture (e.g., Chile), assemble a local panel of thought leaders in the target population, and then create a set of culturally adapted recommendations with tactical components to adopt the DBCD model in that target population [13] (Figure 1).

### Transcultural adaptations of the DBCD model in Chile: the evidence base

A range of studies across Chile highlight the significant prevalence and risk factors associated with DBCD (Table 2). T2D prevalence varies from 1.5% in rural Aymara populations [24] to 17.3% in urban elderly populations [25]. Cognitive impairment is notably higher in older adults with T2D [25]. Insulin resistance, influenced by lifestyle factors and urbanization, shows significant variability between indigenous Mapuche and European populations [26]. Low physical activity, especially in women, has been reported [27]. Drug coverage is incomplete and inequitable [28]. T2D risk is linked to obesity, inactivity, and low education [29]. Cardiovascular programs exhibit insufficient contact but feasible telephone support [30]. In fact, telemonitoring prevents deterioration in glycemic control in Chile [31].

**Table 2:** Dysglycemia-Based Chronic Disease Evidence: Survey of Chilean Evidence.

AUTHOR, YEAR (REFERENCE)	N	GEOGRAPHY	POPULATION	ENDPOINT(S)	RESULTS
Santos et al., [24]	196	Rural Andean provinces (>2,000 m altitude, northern Chile)	Adults >20 years old	Prevalence of cardiometabolic traits	Prevalence: T2D: men 1.3%, women 1.7%. IGT: men 2.6%, women 4.3%. Obesity: men 12.8%, women 23.5%. High total cholesterol (≥ 200 mg/dl): men 36.8%, women 37.4%. Hypertension: men 19.2%, women 17.7%. Sedentariness: men 5.1%, women 3.4%.
Bozanic et al., [25]	358	6 cities	Adult 65 years old (from Diabetes and Dementia project)	Risk factors, prevalence, and association of cognitive impairment and T2D	T2D prevalence: 17.3% Higher prevalence of cognitive impairment with vs. without T2D: 30.7% vs. 13.9% Risk of cognitive impairment: 2.8 times higher with vs. without T2D
Celis-Morales et al., [26]	472	Los Ríos, Bio-Bio, and Metropolitana	Adults 20–60 years old	Impact of environmental/ethnicity factors on lifestyle markers	HOMA-IR (significant ethnicity x environment interaction: *P* = 0.0003): ◦ Rural Mapuche: 1.65 ± 2.03 ◦ Urban Mapuche: 4.90 ± 3.05 ◦ Rural European: 0.82 ± 0.61 ◦ Urban European: 1.55 ± 1.34 The effect of urbanization on HOMA-IR was greater in Mapuche compared to European individuals Ethnicity (significant interactions for all *P* < 0.004): ◦ Adiposity on HOMA-IR ◦ Sedentary time on HOMA-IR ◦ PA on HOMA-IR The effects of adiposity, sedentary time, and PA on HOMA-IR were greater in Mapuche compared to European individuals
Arteaga et al., [27]	983	Limache, Valparaiso	Adults 22-28 years old	PA and cardiovascular risk factors	PA levels: ◦ Men: 3731 ± 3923 METs-minutes/week ◦ Women: 1360 ± 2303 METs-minutes/week Insufficient PA levels: ◦ 50% of women ◦ 21.5% of men Intense PA levels: ◦ 60% of men ◦ 23.4% of women Inverse association PA and insulin resistance High PA was protective against low HDL-c and high TGs, particularly among men
Lange et al., [31]	60 (INT) 681 (CON)	Santiago	Adults 20-64 years old (in Cardiovascular Health Program and with T2D not on insulin)	Efficacy of a telecare self-management support model on metabolic control	A1C levels: ◦ INT: 8.3 ± 2.3% to 8.5 ± 2.2% (maintained, *P* = NS) ◦ CON: 7.4 ± 2.3% to 8.8 ± 2.3% (deteriorated, *P* < 0.001) Perception of self-efficacy: ◦ INT: improved, *P* < 0.001 ◦ CON: unchanged (*P* = NS) INT and CON: no change in adherence to medication, PA, and foot care INT: increased attendance with clinic visits and decreased emergency care visits
Matute et al., [28]	6,233	National	Persons 15 years old from the National Health Survey (2016–2017).	Medication use and effective coverage for T2D, dyslipidemia and hypertension in Chile, considering sociodemographic variables and SDOH	T2D prevalence: 12.3% (majority were women 58.1%) Beneficiaries of the Public Health System (FONASA): 80.9% Medication’s use among patients with diabetes: 60.7% ◦ Higher medication’s use in women ◦ Lower medication’s use in younger age groups <65 years old ◦ Lower medication’s use among indigenous peoples ◦ Medication use increased with more years of study With effective coverage for diabetes management, 54.2% had their disease controlled
Piette et al., [30]	569	Puente Alto, Santiago	Adults 30-75 years old in CVD program	Patient characteristics and feasibility of extending reach with structured nurse telephone contacts between outpatient encounters	Medications: ◦ Oral agents 79%; metformin 58%; glyburide 65%; tolbutamide 8% ▪ Using one oral agent: 49% ▪ Using two oral agents: 40% ◦ Insulin: 3% CVD program visits: ◦ Met the target of two visits in the past 6 months: 33% ◦ Made more than three visits: 32% ◦ Inadequate program contact: 27% (many in poor health) Program impact: ◦ Greater use of CVD program associated with higher patient satisfaction (after controlling for confounders) ◦ Many participants had difficulties with lifestyle changes ◦ Greater use of CVD program not associated with healthier behaviors Telephone access: ◦ Reported telephone access: 95% ◦ Used telephone to contact clinic: 37% ◦ Majority willing to use telephone care for behavior change and emotional support
Leiva et al., [29]	4700 (538 with and 4,162 without T2D)	National	Subjects > 15 years old from National Health Survey (2009–2010)	Associations of T2D with SDOH and lifestyle factors	Subjects with vs. without T2D had lower educational level, lower SES, and higher BMI and WC Subjects with T2D reported: ◦ Lower total PA ◦ Greater consumption of salt, fruits, and vegetables ◦ Higher overall dietary score ◦ Higher prevalence of ex-smokers or non-smokers ◦ Poor to fair perception of their health/wellness. ◦ Increased metabolic complications ◦ Higher incidence of family history of T2D Greater risk for T2D with: ◦ Overweight/obesity ◦ Sleep > 9 hours nightly ◦ Physical inactivity ◦ Hypertension Lowed risk for T2D with: ◦ Intermediate or higher educational level ◦ Good perception of well-being/health

These data are used for transculturalizing DBCD recommendations. Since only significant ORs were reported, 95%CI was omitted for simplicity.

*Abbreviations:* A1C—hemoglobin A1c, BMI—body mass index; CI—confidence interval, CVD—cardiovascular disease, CON—control group, DBCD—dysglycemia-based chronic disease; FBG—fasting blood glucose; HCP—healthcare professional; HDL-c—high-density lipoprotein cholesterol, HOMA-IR—Homeostatic Model Assessment for Insulin Resistance, IGT—impaired glucose tolerance, INT—intervention group, MET—metabolic equivalent of physical activity, OR—odd ratio, PA—physical activity, SDOH—social determinants of health; SES—socioeconomic status, T2D—type 2 diabetes; TGs—triglycerides; WC—waist circumference. Values expressed as mean standard deviation.

Table 3 summarizes Chilean studies highlighting transcultural lifestyle influences and DBCD-related determinants across biological, behavioral, and social domains, including genetics, nutrition, physical activity, and socioeconomic factors. Genetically, *SLC16A11* risk variants are frequent and independently associated with higher body mass index (BMI) in adults without T2D [32]. Studies on health literacy show that most patients and HCPs view obesity as a chronic disease, yet patients largely individualize responsibility and report structural and economic barriers to weight control [33]. University and national surveys document sedentariness, unhealthy diets, excess weight, and obesity that co-occur with T2D, hypertension, and dyslipidemia, especially in low-SES and indigenous groups [34–36]. Physical inactivity and prolonged sitting independently predict T2D, obesity, hypertension, metabolic syndrome, and central adiposity [37–39]. Additional contributors include newly defined insulin resistance and WC cut-points for risk stratification [40, 41], as well as the impact of weight stigma on BMI [42].

**Table 3:** Dysglycemia-Related Factors Based on Chilean Evidence.

CATEGORY	AUTHOR, YEAR (REFERENCE)	OBJECTIVE	RESULTS
Genetics	Mardones et al., [32]	Association of SLC16A11 gene variants with obesity and metabolic markers in those without diabetes	263 non-diabetic adults, minor allele of SLC16A11 gene prevalence 29.7% Higher BMI is independently associated with polymorphic SLC16A11 genotypes
Health Literacy	Cuevas et al., [33]	Identify barriers, perceptions, attitudes, behaviors, and barriers in obesity care	Survey completed by 1,000 patients with obesity and 200 HCPs, 74% of patients with obesity and 95% of HCPs view obesity as a chronic disease Most patients with obesity believe they are personally responsible for their weight loss Patients identified lack of exercise and cost of weight management as significant barriers to weight loss
Nutrition	Ratner et al., [34]	Analyze eating behaviors, nutritional status, and history of previous diseases in students of higher education	Among 6823 students 17–29 years old, 47% skipped breakfast and 35% skipped lunch daily Daily consumption of vegetables, fruits, and dairy products was low; consumption of soft drinks, chips, cakes, and sweets was high 76% of students were sedentary, 40.3% were smokers, and 27.4% were overweight/ obese, with the latter group having more diabetes, hypertension, and hypercholesterolemia There was poor agreement between actual nutritional status and self-perception, particularly among males Students with a food scholarship from the Ministry of Education had a higher frequency of eating lunch
	Mujica-Coopman et al., [35]	Assess relationships among malnutrition, SES, and ethnicity	NHS conducted in 2016–2017, over 75% with excess weight Women with lower SES had higher excess weight and shorter stature In men, excess weight did not vary significantly by SES, but short stature was more common with low SES Obesity more frequent in indigenous women and men
	Cediel et al., [36]	Assess the consumption of ultra-processed foods and associations with nutrients related to non-communicable diseases	Chilean population aged ≥2 years (n 4920) from a NHS (2010) Ultra-processed foods are 28.6% of total energy intake Positive association between ultra-processed food consumption and higher intake of nutrients that promote non-communicable diseases (e.g., free sugars, total fats, saturated fats, *trans* fats, and increased Na:K ratio; negative association with protective nutrients (e.g., potassium and fiber) Apart from sodium, inadequate nutrient intake increased with higher consumption of ultra-processed foods Reducing ultra-processed food intake to the lowest quintile could significantly reduce nutrient inadequacy
Physical activity	Celis-Morales et al., [37]	Examine PA and sedentariness prevalences by SES	Chilean NHS (2009–2010), 5434 persons aged ≥15 years (59% women), 19.8% did not meet PA recommendations (≤600 MET min/week) PA was more prevalent among subjects ≥65 years old, and women exhibited higher rates than men Subjects with higher education or income levels showed lower PA The overall prevalence of sedentary behavior (>4 hours sitting per day) was 35.9%
	Díaz-Martínez et al., [38]	Investigating association between self-reported sitting time and diabetes-related markers	Chilean NHS (2009–2010), 4,457 adults aged ≥18 years, the OR for T2D increased by 1.10 for men and 1.08 for women for each additional hour of sitting time, independent of age, education, smoking, BMI, and total PA The prevalence of T2D was 10.2% with lowest sitting time and 17.2% with highest sitting time
	Díaz-Martínez et al., [39]	Investigate the association of PA with obesity, metabolic markers, T2D, hypertension, and metabolic syndrome	Chilean NHS (2009–2010), *n* = 5,157. Inactive men and women had higher ORs (1.77 and 1.25, respectively) for obesity than physically active peers Inactive men and women had higher ORs (2.47 and 1.72, respectively) for T2D than physically active peers Inactive men and women had higher ORs (1.66 and 1.83, respectively) for hypertension than physically active peers An association between physical inactivity and central obesity (OR: 1.92) and metabolic syndrome (OR: 1.74) was observed only in men
Risk Assessment Tools	Arancibia et al., [40]	Assess OGTT serum insulin levels to gauge insulin resistance	Retrospective analysis of 1,815 OGTTs in non-diabetic adults aged 18–75. 75th percentiles for serum insulin levels: 60 minutes - 127 μU/mL; 120 minutes - 81 μU/mL High correlation (*r* = −0.89) between HOMA-IR and composite whole-body insulin sensitivity index
	Petermann-Rocha et al., [41]	Identify sex-specific cut-off points for WC for metabolic syndrome diagnosis	Chilean NHS (2003, 2009–2010, 2016–2017), 8,182 persons aged ≥15 years. Optimal cut-off points for WC defining metabolic syndrome were 92.3 cm for men and 87.6 cm for women
Mental Health	Bastias-González et al., [42]	Determine whether psychological variables and behavioral variables predict obesity	344 adults (55.8% women). Sociodemographic covariates did not significantly predict BMI Physiological covariates, behavioral variables, and weight stigma were associated with BMI Weight stigma was the predictor explaining the most BMI variance

These factors are used for transculturalizing DBCD recommendations.

*Abbreviations:* BMI—body mass index; CI—confidence interval; CON—control group; CPAP—continuous positive airway pressure; DBCD—dysglycemia-based chronic disease; HOMA-IR—homeostatic model assessment for insulin resistance; INT—intervention group; LDL-c—low-density lipoprotein cholesterol; MET—metabolic equivalent of task; NHS—National Health Survey; OGTT—oral glucose tolerance test; OR—odd ratio; PA—physical activity; SES—socioeconomic status; T2D—type 2 diabetes.

### Achieving consensus

The objective of this project was to develop a consensus document outlining affirmed and emergent concepts, key strategies, and then specific implementation tactics to improve T2D care in Chile. More specifically, attention was focused on addressing research gaps (scientific questions without answers), knowledge gaps (answers without awareness), and practice gaps (awareness without action). To achieve this, an international multidisciplinary conference was convened with an expert panel representing four foundational sectors: (1) Biomedical; (2) Education, Research, and Professional Organizations; (3) Healthcare Industry; and (4) Government/Regulatory and Patient Advocacy. This diverse team of stakeholders included researchers, educators, HCPs, and patient representatives, ensuring a diverse set of voices needed for an impactful and relevant discussion on DBCD management in Chile.

## Conference and Review Methods

This consensus document was developed through a structured, multidisciplinary conference designed to generate context-specific affirmed (previously described) concepts, emergent (newly described) concepts, key strategies, and implementation tactics for adapting the DBCD framework to Chile. The structure of this consensus conference was modeled after the 2014 AACE Obesity Consensus Conference [43]. The methodology combined pre-meeting evidence review, structured questionnaire assessment, moderated group deliberation, and post-meeting synthesis. Four key stakeholder pillars were identified as essential for advancing T2D care (Table 4). The Biomedical pillar included organizations represented healthcare teams treating T2D. The Education, Research, and Professional Organizations pillar represented scientific organizations and educational institutions advocating T2D care. The Healthcare Industry pillar represented companies involved in the development of T2D medications, nutritional products, and diabetes technologies. Finally, the Government/Regulatory and Patient Advocacy pillar represented groups responsible for creating healthcare policies and managing population-based T2D prevention and economic considerations.

**Table 4:** METRICS/LSP Consensus Conference on DBCD Transculturalization in Chile—Pillar Participants.

PILLARS	PARTICIPANTS	
Biomedical	Jeffrey I. Mechanick, MD (Chair)	The Marie-Josee and Henry R. Kravis Center for Cardiovascular Health at Mount Sinai Fuster Heart Hospital, Icahn School of Medicine at Mount Sinai, New York, NY, USA; and METRICS, USA
	Ramfis Nieto-Martinez, MD, MSc (Co-Chair; DBCD-CC General Coordinator)	Precision Care Corp, Saint Cloud, FL, USA; Lown Scholar Program, Harvard TH Chan School of Public Health, Boston, MA, USA; FISPEVEN INC, Venezuela; and METRICS, USA
	Carlos Grekin, MD (DBCD-CC Chile Coordinator)	Nutrition and Diabetes Unit, Clínica Red Salud Vitacura; Nutrition and Diabetes Service, Santiago Military Hospital; Universidad de Los Andes, Santiago, Chile; and METRICS, USA
	Diana De Oliveira-Gomes, MD	Division of Cardiovascular Medicine, Brigham and Women’s Hospital, Harvard Medical School, Boston, MA, USA; FISPEVEN INC, Venezuela; and METRICS, USA
	Manuel Moreno, MD, MSc	Pontificia Universidad Católica de Chile. Departamento de Nutrición, Diabetes y Metabolismo, Facultad de Medicina. Santiago, Chile
	Carolina Ceron Reyes, MD, MBA	Centro Médico y Dental. Red Salud Arauco. Santiago, Chile.
	Eduardo Figueroa Psi	Servicio de Neurología. Hospital Militar. Santiago, Chile
	Alex Valenzuela Montero, MD	Facultad de Medicina. Clínica Alemana Universidad del Desarrollo. Nutrición y Dietética, Santiago, Chile.
	Víctor Saavedra, MD	Sociedad Chilena de Obesidad (SOCHOB), Santiago, Chile.
	Claudia Cancino, RD	RedSalud Arauco, Santiago, Chile
Education, Research, and Professional Organizations	Goodarz Danae,i DSc (Keynote speaker)	Director, LSP-Harvard, Boston, MA, USA
	Juan Pablo González, MD (DBCD-CC Online Moderator)	Lown Scholar Program, Harvard TH Chan School of Public Health, Boston, MA, USA; FISPEVEN INC, Venezuela, and METRICS, USA
	Sandra López Arana, PhD, MSc, RD	Universidad Finis Terrae, Escuela de Nutrición y Dietética, Santiago, Chile; Lown Scholar Program, Harvard TH Chan School of Public Health, Boston, MA, USA
	Báltica Cabieses, MSc, PhD	Universidad del Desarrollo, Centro de Salud Global Intercultural (CeSGI), Santiago, Chile
	Guillermo Cortes, MSc	Universidad Viña del Mar. Facultad de Ciencias Jurídicas, Sociales y de la Educación. Escuela de Educación, Viña del Mar. Chile
	Sandra Vesga, MSc	Universidad Viña del Mar. Facultad de Ciencias Jurídicas, Sociales y de la Educación. Escuela de Educación, Viña del Mar. Chile
	Cecilia Albala, MD, MPH	Instituto de Nutrición y Tecnología de los Alimentos (INTA), Universidad de Chile, Santiago, Chile.
	Hernán Speisky Cosoy, PhD	Instituto de Nutrición y Tecnología de los Alimentos (INTA), Universidad de Chile, Santiago, Chile
	Francisco Pérez-Bravo, PhD	Laboratorio de Micronutrientes. Unidad de Nutrición Humana. Instituto de Nutrición y Tecnología de los Alimentos (INTA). Universidad de Chile, Santiago, Chile.
Healthcare Industry	Francisco Javier Smart, PE	Boston Consulting Group. Santiago, Chile
	Arturo Avendaño Bravo, PE	Central de Abastecimiento del Sistema Nacional de Servicios de Salud (CENABAST). Unidad de Inteligencia de Negocios. Santiago, Chile
	Benjamín Medina, PhD	Nova Foods S.A. Santiago, Chile
Government/Regulatory and patient advocacy	Pedro Barria Gutierrez, JD	Unidad de Mediación de Daños en Salud. Consejo de Defensa del Estado. Santiago, Chile
	Claudia Pradenas, Patient	Diabetes Araucania Temuco. Temuco, Chile

*Abbreviations:* DBCD Chile-CC—Consensus Conference on Dysglycemia-Based Chronic Disease (DBCD) Transculturalization in Chile; FISPEVEN INC—Foundation for Clinic, Public Health, and Epidemiology Research of Venezuela; METRICS—The MEchanick Transculturalization Research and Innovation ConSortium.

**Table 5:** Chilean DBCD Recommendations, Key Strategies, and Implementation Tactics

COMPONENT	RECOMMENDATIONS			KEY STRATEGIES	IMPLEMENTATION TACTICS
Risk Screening		Enhance the detection of T2D and CVD risk in the Chilean population using culturally adapted tools.	Validate risk assessment tools, such as FINDRISC, tailored to Chile’s biological, cultural, and socioeconomic context. Use the Chilean version of GLOBORISK as CVD risk score.		Use the tDNA framework to address cultural differences in tool adaptation and validation.^a^
					Involve community leaders to pragmatize these tools, enhancing their accuracy and cultural relevance.^b^
					Conduct studies to assess the validity and implementability of these tools across diverse settings.^c^
Culture and SDOH		Incorporate SDOH and ethnocultural factors to DBCD care.	Collect and integrate socioeconomic and cultural data from Chileans into patient care strategies.		Incorporate SDOH into public policies for inclusive care.^d^
					Utilize community resources to address SDOH.^e^
		Use accessible language and culturally relevant resources during HCP–patient interactions.	Design and embed cultural competence training into HCPs curricula.		Train healthcare teams in culturally sensitive communication.^f^
					Partner with educational institutions and community organizations.^g^
Healthcare Access and Equity		Promote equity in healthcare access.	Ensure equitable access to quality healthcare, especially for vulnerable populations.		Develop initiatives like PIAAM to integrate eHealth and comprehensive support for migrants.^d^
					Work with organizations specialized in supporting indigenous populations such as Mapuches.^b^
		Optimize access to clinical practice guidelines recommended medications.	Improve access to effective and affordable T2D treatments.		Establish policies for the provision of key medications.^d^
					Inform professionals about optimized treatment options.^f^
Community Engagement and Education		Community integration and education.	Use community resources to promote education and support health initiatives.		Engage local leaders to strengthen education about prediabetes and T2D.^e^
					Support healthy lifestyle changes with infrastructural resources.^h^
		Education on alcohol consumption risks.	Promote strategies to educate the public about the risks of alcohol consumption.		Include diverse disciplines in alcohol education campaigns.^i^
					Develop policies such as taxation to dissuade unhealthy habits.^d^
		Strengthening community-level nutrition education efforts.	Promote primary prevention by implementing nutrition education initiatives focused on local healthy foods and dietary patterns.		Implement initiatives like “Healthy Food Prescriptions” and school-based programs such as “Kiosco Verde.”^d^
Individualized Care and Recommendations		Individualized nutritional recommendations.	Make dietary recommendations personalized, considering socioeconomic factors.		Align nutritional recommendations with patients’ individual realities. ^j^
					Conduct studies on the effectiveness of personalized diets in various populations.^c^
		Local disease progression understanding.	Understand and address T2D progression in the Chilean context.		Promote longitudinal studies to understand local disease patterns.^c^
					Partner with local institutions to collect relevant data.^g^
Technological and Strategic Integration		Efficient EHR use	Leverage effective use of EHR to improve data quality and comprehensive treatment		Enhance electronic record systems.^k^
					Encourage collaboration to maximize EHR effectiveness.^i^
		Strategic planning and prioritization.	Develop a strategic plan prioritizing actions based on local needs.		Involving key participants in strategic planning.^b^
					Create policies promoting evidence-based local interventions.^d^
Evaluation and Effectiveness		Evidence on cost-effectiveness.	Generate local evidence on the cost-effectiveness of T2D prevention strategies.		Conduct studies to assess cost-effectiveness and guide public health policy.^c^
					Publish documents supporting informed and effective decision-making.^l^
Communication and Language Use		Using appropriate language.	Educate HCPs on the importance of respectful and non-stigmatizing language.		Train in using patient-centered language.^f^
					Ensure language is culturally sensitive and appropriate.^a^

By consensus, affirmed and emergent concepts were used to formulate recommendations, which were then interpreted as specific strategies with respective implementation tactics.

*Abbreviations:* DBCD—dysglycemia-based chronic disease; EHR—electronic health record, HCP—healthcare professional; SDOH—social determinants of health; SES—socioeconomic status, T2D—type 2 diabetes.

Implementation tactics are: transculturalization,^a^ stakeholder engagement,^b^ research,^c^ public policies,^d^ community-based interventions and engagement,^e^ healthcare team education and training,^f^ collaboration and networking,^g^ lifestyle medicine infrastructure,^h^ multidisciplinary teams,^i^ patient-centered approach,^j^ health information technology,^k^ and white papers.^l^

The DBCD Chile Consensus Conference (DBCD Chile-CC) was launched by the MEchanick Transculturalization Research and Innovation ConSortium (METRICS) leadership (https://metricscm.com/). METRICS is a consortium founded in 2014 dedicated to addressing global cardiometabolic risk through transcultural, lifestyle, and precision healthcare adaptations. To enhance public health strategies, an alliance was established with the Bernard Lown Scholars in Cardiovascular Health Program at Harvard T.H. Chan School of Public Health (https://hsph.harvard.edu/fellowship-special-program/lown-scholars/), a program founded in 2008 to train HCPs worldwide in preventing CVDs in developing countries. A METRICS leadership task force planned the conference, beginning with the selection of major stakeholders, including endocrinologists, cardiometabolic specialists, lifestyle experts, administrators, public health professionals, and patient representatives. Invitations were sent to 43 participants, with 32 accepting and 24 attending the meeting, of whom 13 participated in person and 11 attended online.

The goal of the DBCD Chile-CC was to achieve consensus on five essential questions. Pre-meeting activities involved participants familiarizing themselves with these questions before attending the conference. Pre-meeting activities were divided into three phases: to read the proposal drafted by the task force, complete a questionnaire, and prepare their opinions. The pre-meeting questionnaire included 33 structured statements covering screening, diagnostic thresholds, lifestyle interventions, transcultural adaptation, and implementation strategies. Participants rated their level of agreement using a 5-point Likert scale (strongly disagree to strongly agree).

The meeting activities took place on November 20, 2023, in Santiago de Chile. Participants were divided into four groups, and the five consensus questions were distributed among them. Each group included members from all four pillars. The discussions were oriented to specifically answer each question while formulating three recommendations and their respective key strategies and implementation tactics. One member from each group presented their findings in a 10-minute session using oral statements, handouts, or slide presentations. This was followed by a general discussion and debate moderated by the co-chairs, allowing all participants—both in-person and online—to share their perspectives. Final classification of concepts was achieved through open discussion and moderated consensus, rather than anonymous re-voting, reflecting the qualitative nature of consensus-building in implementation science [44].

Proceedings were recorded and transcribed, with a team from the Task Force and conference leadership integrating critical discussion points and conclusions in real time. Post-meeting activities involved a METRICS writing task force comprehensively reviewing the current evidence, survey results, meeting recordings, and conference discussions to develop a draft document. The current evidence comprised peer-reviewed articles of studies published in the databases MEDLINE, SCOPUS, SCIELO until September 2024 and webpages, without restrictions on language. The search was designed using the search terms: “Chile,” “Chilean,” “Diabetes,” “Type 2 Diabetes,” “Dysglycemia,” “Prediabetes,” “Insulin Resistance,” “Transculturalization,” and “Transcultural.” Conference participants from organizing institutions reviewed the draft document and were asked to confirm consensus with the presented concepts.

## Results and Discussion

### Question 1. What constitutes a comprehensive approach to T2D care?

A comprehensive approach to T2D care emphasizes quality metrics addressing the full complement of downstream complications and associated comorbidities, based on patient engagement, precision treatment, multidisciplinary teams, and long-term well-being.

The consensus group concluded that while Chile’s approach to managing T2D includes essential care, important aspects should also be incorporated – implementation, quality, continuous monitoring, and service integration. Electronic health records (EHRs) efficiently collect, track, and analyze patient data, enabling interventions and care coordination. Consistent and accurate data entry enhances care quality and performance. A study conducted at a teaching hospital in Santiago, Chile, which averages 3,000 visits per month, found that the completeness of EHR data was adequate [45]. However, there is no information available on the quality of the data included in the EHR. Reducing bureaucratic burdens frees up time for direct patient care. Effective T2D care requires cross-sector collaboration, integrating health education from kindergarten through primary school. Embedding health concepts in curricula and employing long-term policy-driven strategies beyond political cycles can lead to sustainable improvements.

### Question 2. Why is prediabetes important to diagnose?

Early diagnosis of prediabetes is crucial, as it presents an opportunity to prevent progression to T2D and T2D-related complications, especially CVD. The consensus group emphasized that clinical inertia during the prediabetes stage (i.e., waiting to implement management strategies when diagnostic hyperglycemic criteria for T2D are satisfied) can increase the risk of T2D complications and clinical and economic burdens. Prediabetes can lead to cardiovascular complications without intervening T2D [46]. Identifying prediabetes enables early lifestyle interventions and improves outcomes, not requiring glucose-lowering pharmacotherapy. Given the limitations of diagnostic tools and data, pinpointing the time of prediabetes to T2D transition is difficult. Thus, strategies must focus on prediabetes, not in isolation, but rather as part of a broader DBCD continuum.

A multipronged approach to prediabetes fosters healthy living, early childhood education, and long-term public health policies that exceed short-term goals [47]. Establishing specialized lifestyle medicine centers or service lines for healthy living and proactive health management—starting before birth and involving intersectoral cooperation with primary family healthcare centers (e.g., CESFAM)—can support these long-term objectives [48]. Moreover, CESFAM has served as a framework for designing the transcultural adaptation of a Mental Health intervention program [49]. These centers would monitor health outcomes in specific populations to demonstrate the effectiveness of early interventions and continuous, integrated care. Enhancing the quality and quantity of electronic health data, adopting an integrative approach to managing prediabetes and T2D, and advocating for long-term health policies are essential to improving T2D prevention and management.

### Question 3. Is the diagnosis of DBCD useful in chile and how should screening and case finding be considered?

The consensus group agreed that diagnosing DBCD in Chile is valuable, but several challenges must be addressed. A major concern is the late detection and treatment of dysglycemia, as many individuals live with prediabetes and insulin resistance for up to 15 years before receiving a formal diagnosis. By that time, significant macrovascular and microvascular complications may have already developed, severely compromising patients’ health. Screening populations for elements of DBCD such as abnormal adiposity (as a cause of insulin resistance) or hyperglycemia, or case finding in those with identifiable risks are core aspects of early preventive care.

Current tools such as FINDRISC, which detects/predicts 10-year T2D risk provides a useful framework but requires adaptation for the Chilean population. For instance, WC cut-offs should be revised to account for ethnic diversity and demographic changes influenced by migration. The group highlighted the need to adapt and validate FINDRISC and align with local demographics, lifestyle habits, and socioeconomic realities. In the meantime, LA-FINDRISC—a Latin American adaptation incorporating region-specific WC cut-offs (≥94 cm for men and ≥90 cm for women)—is available for use [4]. Beyond WC, BMI thresholds used in Chile may not accurately represent risk across different population groups, underscoring the need for more inclusive criteria. Additionally, physical activity plays a crucial role in T2D risk assessment, yet current screening protocols may fail to capture real-world behavior. As an example, the standard guideline of 30 minutes of exercise, three times per week, may not adequately reflect varying activity patterns across age groups, particularly in children who engage in spontaneous daily movement. Given these factors, the group agreed on the importance of initiating screening at younger ages, especially for individuals with familial risk factors such as parental obesity or T2D.

Current diagnostic tools need refinement based on national data and population characteristics. Case-finding for disease in patients already at risk should extend beyond glycemic control to include lipid levels, blood pressure, and physical activity. Socioeconomic and sociocultural factors must also be considered. Early detection of risk, predisease, disease, and even complications should be part of a broader strategy centered on education and prevention. Community initiatives, patient education, and HCP training are also important to close/narrow knowledge gaps in DBCD care. The consensus group emphasized the need for developing and implementing a transcultural diagnostic algorithm validated within the Chilean context. This effort will require collaboration among policymakers, HCPs, scientists, and community leaders.

### Question 4: What are the main features of a chilean transcultural approach to DBCD?

The consensus group agreed that a Chilean transcultural approach to DBCD management embraces the following important features:

- Understanding the current population composition, including ethnocultural diversity and migration trends in Chile, which must be explicitly delineated to design interventions that address unique needs and ethnocultural contexts;
- Tailoring implementation tactics to socioeconomic and sociocultural contexts to ensure acceptability and affordability;
- Adapting risk assessment tools such as FINDRISC to reflect Chilean demographic variations, including specific WC and BMI thresholds;
- Designing culturally sensitive health programs for specific ethnic groups, including indigenous populations such as the Mapuche – this involves understanding and integrating lifestyle and health practices into preventive and therapeutic decision-making;
- Create practical and sustainable healthcare strategies through stakeholder engagement (e.g., patients, HCPs, policymakers, and industry representatives);
- Empowering patient advocacy groups and diabetes-related scientific societies to promote health education and adherence to management plans;
- Developing and then adapting evidence-based therapeutic and preventive clinical practice guidelines to real-world Chilean factors, with subsequent iterative optimization processes; and
- Securing financial backing from legislative bodies to provide policy continuity, economic support, and long-term impact beyond political cycles.

### Question 5: What are the core Chilean lifestyle interventions for each DBCD stage?

The consensus group agreed that the core lifestyle interventions for DBCD stages and CVD in Chile should focus on three main aspects: dietary changes, physical activity, and education.

#### Dietary interventions

Programs must be implemented for patients with diet-related health risks, food insecurity, or other challenges when accessing nutritious foods. In this preventive service, HCPs can prescribe fruits, vegetables, and other healthy foods, which patients—especially those from lower socioeconomic backgrounds—can redeem at participating stores. Agencies such as the National Board of School Aid and Scholarships (Junta Nacional de Auxilio Escolar y Becas; JUNAEB) must be explored and utilized to provide financial support to subsidize the purchasing of healthy food [50]. This could include specific vouchers or credits accepted at supermarkets for fruits and vegetables. Educational programs must be developed to teach patients the importance of proper nutrition and healthy meal preparation. This messaging approach is part of the Produce Prescription program and is also supported by T2D and various professional medical/scientific societies that focus on nutrition.

#### Physical activity

“Exercise is Medicine” and related approaches must be adopted whereby HCPs prescribe specific exercise regimes similar to how medications are prescribed. An Exercise is Medicine National Center was established in Chile in August 2018 [51]. Collaboration with local governments is necessary to create more public spaces for exercise and promote community-based physical activity programs. Children, especially those in higher risk groups, must have ample opportunities for physical exercise in schools.

#### Education

Nutrition and health education must be embedded into school curriculum from an early age to instill good habits. Programs such as “Kiosco Verde” in schools can teach children about healthy eating and positively influence family habits. Education programs related to DBCD for patients and their families must be available. These programs would cover diet, exercise, and overall lifestyle changes and utilize patient associations to disseminate information and provide support. Social media, digital tools, and their applications must be leveraged to promote health education and broadly disseminate healthy lifestyle information. Partnering with influencers can enhance the reach and impact of these messages.

### Recommendations, key strategies, and implementation tactics

Consensus recommendations that formulate key strategies and implementation tactics were derived from participants’ discussions of the five guiding questions (Table 5). The panel’s strategy for adopting the DBCD model in Chile highlights the critical role of transcultural adaptation. Validating T2D risk assessment tools tailored to the Chilean bio-sociocultural context, promoting stakeholders’ collaboration, engaging community leaders, and conducting research to ensure their accuracy and relevance across diverse populations is essential. One important part of this strategy is the integration of SDOH and ethnocultural factors into the DBCD care framework. Public policies must reflect these factors to enable inclusive healthcare delivery. The panel proposed exploring community-based interventions that address the root causes of health disparities. Supporting initiatives such as “Healthy Food Recipes” and training healthcare teams in culturally sensitive communication can improve the use of accessible language and culturally relevant resources in Chile, enhancing patient engagement and adherence [52].

Achieving equitable healthcare access for marginalized groups—such as the elderly, low-income individuals, indigenous communities, and migrants—remains a central priority. The panel recommends developing public policies that integrate eHealth and comprehensive support systems, including programs such as the Interdisciplinary Program for the Care and Integration of Migrants [53]. Additionally, improving access to medications requires evidence-based policies and targeted training for HCPs to support informed decision-making and help patients navigate the system. This comprehensive and culturally responsive approach addresses current gaps while establishing a sustainable framework for effective diabetes care tailored to Chile’s unique sociocultural context.

### Opportunities and challenges in adapting the DBCD model in Chile

Although the transcultural adaptation of the DBCD model in Chile presents important opportunities, the consensus panel acknowledged persistent knowledge gaps in T2D management and prevention. Additionally, implementation challenges must be addressed to narrow practice gaps. A critical factor for success is strong stakeholder engagement and cross-sector collaboration among HCPs, policymakers, community organizations, and government to ensure culturally sensitive and equitable healthcare access.

Important barriers include limited resources, varying levels of health literacy, and systemic resistance to change. Committed integration of SDOH into routine clinical care requires a paradigm shift, which may encounter institutional obstacles and demands for extensive HCP training. Furthermore, equitable access to healthcare and medications requires both policy innovation and solutions to logistical hurdles, such as drug distribution and affordability—especially in rural and underserved areas. Research gaps must also be addressed, including generating local evidence on the cost-effectiveness of proposed interventions and developing adaptable, sustainable strategies aligned with Chile’s evolving healthcare needs.

## Conclusions

A Consensus Conference in Chile identified affirmed and emergent concepts in T2D care, incorporating transcultural insights from the opinion leaders and experts with experience in treating Chilean patients. A set of key strategies and implementation tactics for improving dysglycemia management has been generated from this Consensus Conference. Participants agreed that the DBCD model, by integrating cultural and social aspects into T2D care, enables the early activation of preventive interventions, reducing both disease and economic burdens in Chile. Furthermore, the model is well-suited for transcultural adaptation. The next steps should focus on implementing specific pilot strategies within the Chilean healthcare system. From an aspirational standpoint, this templated methodology for organizing and conducting the conference and generating this set of pragmatic strategies and tactics can be applied to any region in the world. In fact, lessons learned from each transculturalized version of this process can not only inform but also enhance subsequent efforts.
